# Realized Heritability and Fitness Costs of Diflubenzuron Resistance in *Musca domestica* L. (Diptera: Muscidae)

**DOI:** 10.3390/insects17050480

**Published:** 2026-05-08

**Authors:** Abdulwahab M. Hafez, Naeem Abbas

**Affiliations:** Pesticides and Environmental Toxicology Laboratory, Department of Plant Protection, College of Food and Agricultural Sciences, King Saud University, P.O. Box 2460, Riyadh 11451, Saudi Arabia

**Keywords:** house fly, insect growth regulator, two-sex life table, demographic traits, population parameters

## Abstract

The house fly, *Musca domestica* L., is a medically important cosmopolitan pest distributed worldwide. Diflubenzuron, a chitin synthesis-inhibiting insect growth regulator, is widely used for the control of medically important insect pests. In this study, the biological and population characteristics of diflubenzuron-unselected (Diflu-Unsel) and diflubenzuron-selected (Diflu-Sel) *M. domestica* were evaluated using the age–stage, two-sex life-table theory to support resistance management efforts. The results showed that the Diflu-Sel (G_46_) strain developed 319.935-fold resistance to diflubenzuron after 42 generations of selection compared with the Diflu-Unsel (G_46_) strain. Using the LC_50_ of diflubenzuron in the Diflu-Unsel (G_5_) as the parental generation before selection, the realized heritability (*h^2^*) of diflubenzuron resistance was estimated as 0.054. Larval and pupal development durations and the total preoviposition period were significantly prolonged, whereas adult duration, total longevity from egg to adult, oviposition period, and fecundity were significantly reduced in the Diflu-Sel (G_46_) strain compared with the Diflu-Unsel (G_46_) strain, resulting in a relative fitness value of 0.39. Population parameters, including the intrinsic rate of increase, finite rate of increase, generation time, doubling time, gross reproductive rate, and net reproductive rate, were significantly lower in the Diflu-Sel (G_46_) strain than in the Diflu-Unsel (G_46_) strain. These results demonstrate that resistance to diflubenzuron can evolve in *M. domestica* but may be accompanied by substantial fitness costs, which may facilitate resistance management. The findings provide valuable insights for designing sustainable diflubenzuron resistance management strategies for *M. domestica*.

## Simple Summary

The house fly (*Musca domestica* L.) is a cosmopolitan medical pest distributed worldwide. Diflubenzuron, a chitin synthesis-inhibiting insect growth regulator, is commonly used to manage medically important insect pests. In this study, the life-history traits of diflubenzuron-unselected (Diflu-Unsel) and diflubenzuron-selected (Diflu-Sel) house fly strains were evaluated by the age–stage, two-sex life-table approach, which is essential for developing effective resistance management strategies. The results showed that the Diflu-Sel (G_46_) house fly strain developed 319.935-fold resistance to diflubenzuron after 42 generations of selection, compared with the Diflu-Unsel (G_46_) strain. Using the lethal concentration 50 (LC_50_) of diflubenzuron in the Diflu-Unsel (G_5_) as the parental generation before selection, the realized heritability of diflubenzuron resistance was estimated as 0.054. Larval and pupal development durations, as well as the total preoviposition period, were substantially prolonged in the Diflu-Sel (G_46_) strain compared with the Diflu-Unsel (G_46_) strain. However, adult longevity, oviposition period, and adult duration were significantly reduced in the Diflu-Sel (G_46_) flies compared with the Diflu-Unsel (G_46_) flies. Fecundity was also markedly reduced in the Diflu-Sel (G_46_), yielding a relative fitness of 0.39. These findings indicate that field populations of *M. domestica* can develop resistance to diflubenzuron. However, this resistance may be associated with substantial fitness costs, suggesting that resistance may be manageable. This information provides a foundation for developing integrated resistance management strategies for the house fly.

## 1. Introduction

The house fly, *Musca domestica* L. (Diptera: Muscidae), a globally distributed pest of livestock and human dwellings, is known to transmit approximately 200 pathogens associated with serious human and animal diseases, including bovine respiratory disease, hepatitis, tuberculosis, avian influenza, diarrhea, poliomyelitis, typhoid fever, and cholera [1,2,3,4,5,6]. Pathogen transmission occurs primarily through direct physical contact with animals, food, and humans [7]. Management strategies for *M. domestica* include chemical control, sanitation, and biological control. However, chemical insecticides remain the most widely used approach due to their rapid knockdown and high efficacy [2].

Diflubenzuron belongs to the benzoylurea class of insect growth regulators (IGRs) and disrupts chitin biosynthesis, resulting in incomplete molting and lethal developmental abnormalities in insects [8]. It is considered highly selective and exhibits low toxicity to non-target organisms [9]. Diflubenzuron has been widely used in public health and livestock systems for the control of medically important pests, including *M. domestica* [10,11]. In livestock environments, diflubenzuron is commonly applied by spraying or incorporating it into manure substrates [12,13]. In Saudi Arabia, diflubenzuron is extensively used for controlling medical pests in both human and livestock settings [2,10,14,15]. However, intensive and prolonged use of diflubenzuron has resulted in resistance development, increased control costs, and potential environmental concerns [2,14]. Resistance to diflubenzuron has been reported in *Lucilia cuprina* (Wiedemann) [16], *M. domestica* [2], *Culex pipiens* L. [17,18], and *Bovicola ovis* (Schrank) [19].

Realized heritability (*h*^2^) is a quantitative measure derived from laboratory-imposed selection pressure and is used to predict the extent of genetic variation associated with insecticide resistance in insect pest populations [20,21,22]. Estimating *h*^2^ is therefore critical for developing rational resistance management strategies before resistance becomes widespread in field populations. The *h*^2^ has been previously estimated for several insecticide-resistant strains of *M. domestica* [22,23,24,25,26,27,28,29].

Assessing the fitness cost associated with insecticide resistance through laboratory selection experiments is necessary for the development of proactive, effective management strategies [30,31]. Fitness cost affects the rate at which insecticide resistance increases and the occurrence of outbreaks in pest populations [32,33]. In general, insecticide-resistant insects incur higher biological costs and physiological disadvantages, making them less fit than their susceptible counterparts. Even in the absence of insecticide exposure, the emergence and spread of insecticide resistance may be prevented if resistant pests reveal dominant fitness costs [34]. The fitness costs associated with resistance to different insecticides have been evaluated in agricultural and urban pests, including *M. domestica* [33,34,35,36,37,38,39,40], *Aedes aegypti* L. [41,42], *Plutella xylostella* (L.) [43], *Leucoptera coffeella* (Guérin-Méneville) [44], *Dysdercus koenigii* (Fabricius) [45,46], *Culex quinquefasciatus* Say [47], and *Spodoptera litura* L. [30,48]. The fitness costs linked with resistant alleles may result in resistant pest strains exhibiting disadvantageous physiological and population characteristics relative to insecticide-susceptible pests when insecticides are absent [32,34,49]. This situation can facilitate a reversion from resistance to susceptibility in response to insecticide selection pressure. Understanding this process is therefore critical for effectively managing pest resistance.

An age–stage, two-sex life-table analysis is a widely used and robust protocol for determining the fitness costs of resistance in insecticide-resistant insect pests [50,51]. Previously, Hafez [10] evaluated the risk of diflubenzuron resistance and cross-resistance in diflubenzuron-selected *M. domestica* after 24 generations of selection. However, the fitness cost of diflubenzuron resistance has not been explored in *M. domestica*. Therefore, in the current study, we compared the life-history traits of diflubenzuron-selected (Diflu-Sel, G_46_) and diflubenzuron-unselected (Diflu-Unsel, G_46_) *M. domestica* to assess possible fitness costs using the age–stage, two-sex life-table framework. In addition, by using the Diflu-Unsel (G_5_) as the baseline population, we estimated the realized heritability (*h*^2^) of diflubenzuron resistance in the Diflu-Sel (G_46_) after 42 generations of selection. These findings will inform our understanding of the nature of diflubenzuron resistance and support the development of effective resistance management strategies in *M. domestica*.

## 2. Materials and Methods

### 2.1. Rearing Protocol

Approximately 200 adult *M. domestica* were collected from a dairy farm in Al-Washlah, Riyadh, Saudi Arabia (24.39° N, 46.66° E), using plastic collection jars (19 × 33 cm). Following collection, adults were transferred to transparent cages (40 × 40 cm) in the laboratory and maintained according to protocols described in our previous studies [2,10]. Adults were provided ad libitum with deionized water via a ~3 cm cotton wick and fed a dry diet consisting of powdered milk (1 g, Almarai Company, Riyadh, Saudi Arabia) and granulated sugar (1 g, Al-Osra Company, Jeddah, Saudi Arabia), supplied separately in plastic Petri dishes (9 cm diameter). The adult diet was replaced every two days.

Larvae were reared on a diet composed of wheat bran (40 g, Second Milling Company, Riyadh, Saudi Arabia), yeast (10 g, S.I. Lesaffre, Marcq-en-Barœul, France), dry milk powder (3 g), and sugar (3 g), mixed with deionized water to form a semi-solid paste. The larval diet was placed in 500 mL plastic cups, which were introduced into the adult cages for oviposition and larval development. Egg-containing cups were removed daily and covered with cloth to prevent larval escape. Once the diet was fully consumed, larvae were transferred to 1000 mL glass beakers containing fresh larval diet. Larvae pupated within the beakers, which were subsequently placed inside the cages to allow adult emergence and continuation of the colony. All developmental stages were maintained at 27° ± 2 °C, 65 ± 5% relative humidity, and a 12:12 h light/dark photoperiod.

### 2.2. Development of Diflubenzuron-Unselected and Diflubenzuron-Selected M. domestica Strains

The field-collected *M. domestica* population (G_1_) was reared in the laboratory for five generations. At the fifth generation (G_5_), the Diflu-Unsel was bioassayed, and the LC_50_ for this strain was used as the parental generation before selection to calculate the realized heritability of diflubenzuron resistance in the Diflu-Sel (G_46_). At G_5_, the population was divided into two strains: a diflubenzuron-unselected counterpart strain (Diflu-Unsel) and a diflubenzuron-selected strain (Diflu-Sel). The Diflu-Unsel strain was reared for 46 consecutive generations (G_1_–G_46_) without chemical exposure and was used to calculate the resistance ratio and compare life-table parameters. The Diflu-Sel strain was subjected to continuous selection with diflubenzuron for 42 generations (G_5_–G_46_). Each generation was screened at diflubenzuron concentrations of 0.86–8 mg/L, based on sufficient larval survival (average 50%) to produce the next generation. In each selected generation, approximately 2000 second-instar larvae were exposed to diflubenzuron using a diet-incorporation method as described by Hafez [10]. Adults emerging from surviving pupae were transferred to clean cages to establish the subsequent generation. Both strains were maintained under the same laboratory conditions described above.

### 2.3. Concentration–Response Bioassays with Diflu-Sel (G_46_) and Diflu-Unsel (G_46_) Strains to Determine Resistance Ratio

Diflubenzuron (Diflon^®^ 250 WP, Saudi Delta Company, Riyadh, Saudi Arabia) was used for both laboratory selection and toxicity bioassays. Larval susceptibility to diflubenzuron was evaluated using a diet-incorporation bioassay following previously established methods [2,10]. Five concentrations producing mortality between >0% and <100% were prepared by serial dilution in deionized water. Test concentrations ranged from 0.0078125 to 0.125 mg/L for Diflu-Unsel (G_46_) and 1 to 16 mg/L for Diflu-Sel (G_46_).

For each concentration, 72 mL insecticide solution was thoroughly incorporated into the larval diet (wheat bran 20 g, yeast 5 g, dry milk powder 1.5 g, sugar 1.5 g). Each treatment was replicated three times. A total of 150 second-instar larvae were used per bioassay (30 larvae per concentration, 10 larvae per replicate). Control larvae were treated with deionized water only (30 larvae total; 10 per replicate). All treated larvae were maintained under standard laboratory conditions (Section 2.1) until adult emergence. Mortality was recorded after three weeks post-exposure, and pupae that failed to emerge as adults were recorded as dead.

### 2.4. Realized Heritability (h^2^) Estimation

The LC_50_ for the Diflu-Unsel (G_5_) strain was used to calculate the *h*^2^ of diflubenzuron resistance for the Diflu-Sel (G_46_) strain, and was estimated according to Tabashnik [20]:

(1) $$h^{2}=\frac{R}{S}$$

‘*R*’ is the response to selection for diflubenzuron resistance, calculated as follows:

(2) $$R=\;\frac{log{LC}_{50Diflu-Sel\left(G46\right)}-\log{LC}_{50Diflu-Unsel(G5)}}{N}$$

‘N’ represents the number of generations subjected to selection with diflubenzuron.

‘*S*’ is the selection differential, calculated using the following equation:

(3) S=i×σp

‘*i*’ is the selection intensity, calculated according to the following equation [52]:

(4) i =1.583−0.0193336p+0.0000428p2 + 3.65194/p

‘*p*’ is the percentage survival of the Diflu-Sel (G_46_) strain after 42 generations of selection with diflubenzuron.

‘*σp*’ is the phenotypic variance and was assessed by the following equation:

(5) $$\sigma p\;={\left[0.5(Initial\;Slope+Final\;Slope)\right]}^{-1}$$

### 2.5. Life-Table and Population Trait Assessment

To evaluate the fitness costs associated with diflubenzuron resistance, the G_46_ generation of both the Diflu-Unsel and Diflu-Sel strains was used to assess life-table and population parameters. A total of 150 newly hatched 1st-instar larvae from each of the Diflu-Unsel and Diflu-Sel strains were randomly selected for the fitness-cost experiment. The experiment for each strain included three replicates, each consisting of 50 larvae.

The experimental larvae were transferred to glass beakers (1000 mL) containing an artificial larval diet. The beakers were tightly wrapped with muslin to prevent larval escape, and larval development through pupation occurred within these containers. Survival rates, larval duration, and pupal duration were recorded throughout development.

To assess fecundity, newly emerged males and females (<24 h old) from each Diflu-Unsel and Diflu-Sel strain were paired individually in plastic jars (15 cm × 11 cm). Each pair was considered a replicate for both strains and was fed the diet described in Section 2.1. After two days of pairing, the larval diet was placed in a Petri dish within each jar to facilitate oviposition.

Petri dishes containing eggs were replaced daily, and eggs were counted using a fine hairbrush. The experiment was conducted under the same controlled laboratory conditions described above. The age–stage, two-sex life-table parameters were calculated using equations previously described in our studies [2,45,46]; a detailed description is provided in Table 1.

### 2.6. Bioassay and Life-Table Data Analyses

POLO PLUS software Version 1.0 [58] was used for Probit analysis to obtain LC_50_ values for diflubenzuron, along with their fiducial limits (FLs), chi-square (χ^2^) values, and slopes ± standard errors (SEs). The following equation was used to calculate the resistance ratio (RR):

$$RR=\frac{{LC}_{50}\;of\;diflubenzuron\;in\;the\;Diflu-Sel(G_{46})}{{LC}_{50}\;of\;diflubenzuron\;in\;the\;Diflu-Unsel(G_{46})}$$

Confidence limits (CLs, 95%) for RRs were calculated following Robertson et al. [59]. The RRs were considered significantly different when their CLs did not include the value 1.

The TWO-SEX-MSChart Program Version 2019 [60], with 100,000 bootstrap resamplings, was used to estimate the means ± SEs of life-table and population parameters. Differences between Diflu-Sel (G_46_) and Diflu-Unsel (G_46_) strains were evaluated using paired bootstrap tests, and parameters were considered significantly different when the confidence intervals (CIs) did not include 0 at *p* ≤ 0.05 [51,61,62].

## 3. Results

### 3.1. Diflubenzuron Resistance in Diflu-Sel M. domestica

Under laboratory conditions, continuous selection of the *M. domestica* strain with diflubenzuron for 42 generations resulted in a marked increase in resistance (319.935-fold) compared with the Diflu-Unsel (G_46_) (Table 2).

### 3.2. Realized Heritability (h^2^) of Diflubenzuron Resistance

By using the Diflu-Unsel (G_5_) as the baseline population, the *h*^2^ of diflubenzuron resistance was estimated as 0.054 in the Diflu-Sel (G_46_) (Table 3).

### 3.3. Developmental Durations and Reproductive Parameters of the Diflu-Unsel (G_46_) and Diflu-Sel (G_46_)

The adult preoviposition period (APOP) and female ratio did not differ significantly between Diflu-Sel and Diflu-Unsel. However, the durations of the larval and pupal stages and the total preoviposition period (TPOP) were significantly prolonged in Diflu-Sel compared with Diflu-Unsel. Adult duration, preadult duration, total longevity from egg to adult, and oviposition period were markedly shorter in Diflu-Sel than in Diflu-Unsel. In addition, fecundity was substantially lower in Diflu-Sel than in Diflu-Unsel (Table 4).

### 3.4. Population Parameters and Relative Fitness of Diflu-Unsel (G_46_) and Diflu-Sel (G_46_)

Population parameters, such as intrinsic rate of increase (*r*), finite rate of increase (*λ*), generation time (T), doubling time (DT), gross reproduction rate (GRR), and net reproductive rate (*R*_0_), were markedly reduced in Diflu-Sel when compared with those of Diflu-Unsel. Compared with Diflu-Unsel, the relative fitness value of Diflu-Sel was 0.39 (Table 5).

### 3.5. Age–Stage-Specific Survival Rate (s_xj_), Fecundities (f_x_, m_x_), Maternity (l_x_m_x_), Life Expectancy (e_xj_), and Reproductive Values (v_xj_) in Diflu-Sel (G_46_) and Diflu-Unsel (G_46_)

**Figure 1:**
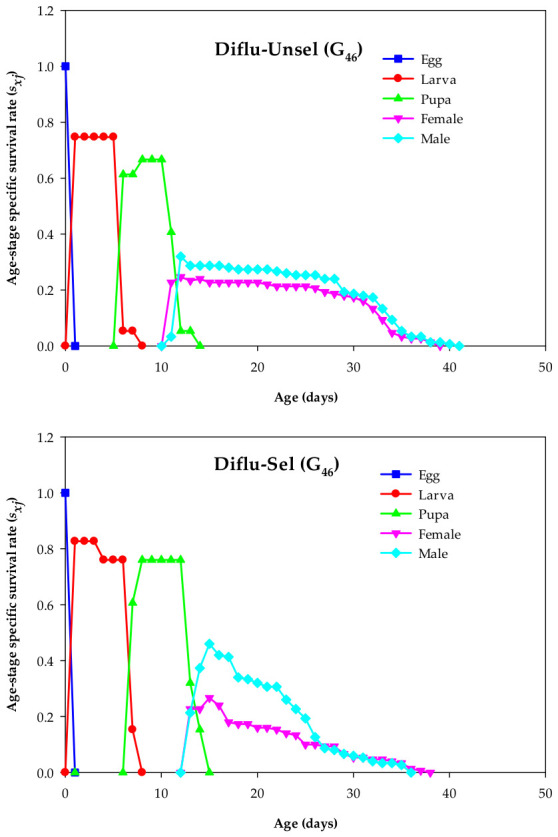
Age–stage-specific survival rate (*s_xj_*) in the diflubenzuron-selected (Diflu-Sel, G_46_) and diflubenzuron-unselected (Diflu-Unsel, G_46_) *Musca domestica*.

*s_xj_* denotes the probability that an individual (as a newly laid egg) survives from the Diflu-Sel and Diflu-Unsel strains to age x and develops to stage j. The maximum *s_xj_* value for the eggs did not differ significantly between the Diflu-Unsel and Diflu-Sel. Nevertheless, the maximum *s_xj_* values for the larvae (0.83), pupae (0.76), females (0.27), and males (0.46) in the Diflu-Sel were higher than those for the larvae (0.75), pupae (0.67), females (0.25), and males (0.32) in the Diflu-Unsel (Figure 1).

**Figure 2:**
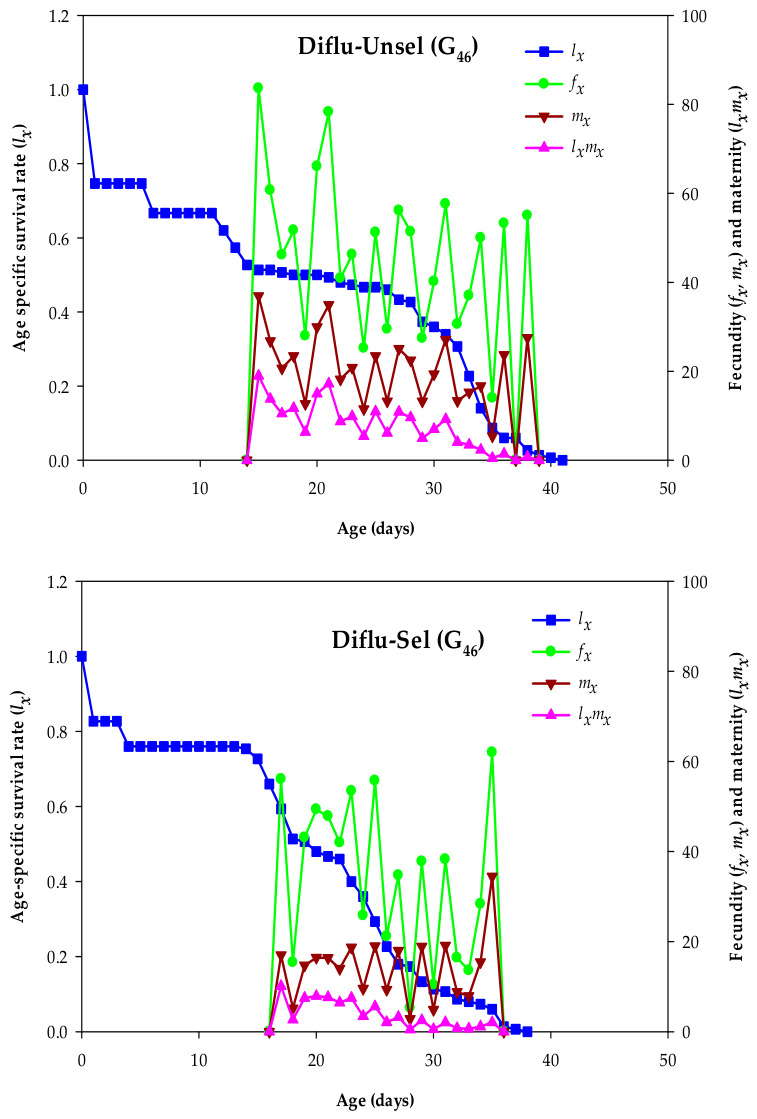
Age-specific survival rate (*l_x_*), age–stage-specific female fecundities (*f_x_*, *m_x_*), and maternity (*l_x_m_x_*) in the diflubenzuron-selected (Diflu-Sel, G_46_) and diflubenzuron-unselected (Diflu-Unsel, G_46_) *Musca domestica*.

The maximum peak values for *l_x_* did not differ substantially between the strains. The highest peaks of *f_x_* (62.00 eggs/female/day) and *m_x_* (34.44 eggs/female/day) were observed in Diflu-Sel females aged 35 days, which were lower than *f_x_* (83.62 eggs/female/day) and *m_x_* (36.92 eggs/female/day) in the Diflu-Unsel females aged 15 days. Similarly, the highest peak value for *l_x_m_x_* in the Diflu-Sel females (10.09 offspring/day at 17 days) was significantly lower than *l_x_m_x_* (18.95 offspring/day at 15 days) in the Diflu-Unsel females (Figure 2).

**Figure 3:**
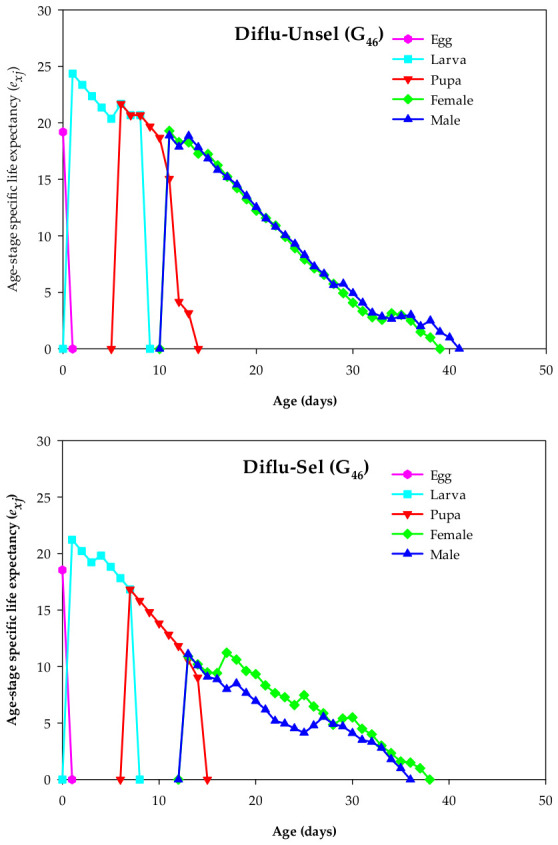
Age–stage life expectancy (*e_xj_*) in the diflubenzuron-selected (Diflu-Sel, G_46_) and diflubenzuron-unselected (Diflu-Unsel, G_46_) *Musca domestica*.

The peak *e_xj_* values in eggs (18.55 days), larvae (21.23 days), pupae (16.82 days), females (11.22 days), and males (11.10 days) of Diflu-Sel were lower than those in eggs (19.19 days), larvae (24.36 days), pupae (21.68 days), females (19.29 days), and males (18.88 days) of Diflu-Unsel (Figure 3).

**Figure 4:**
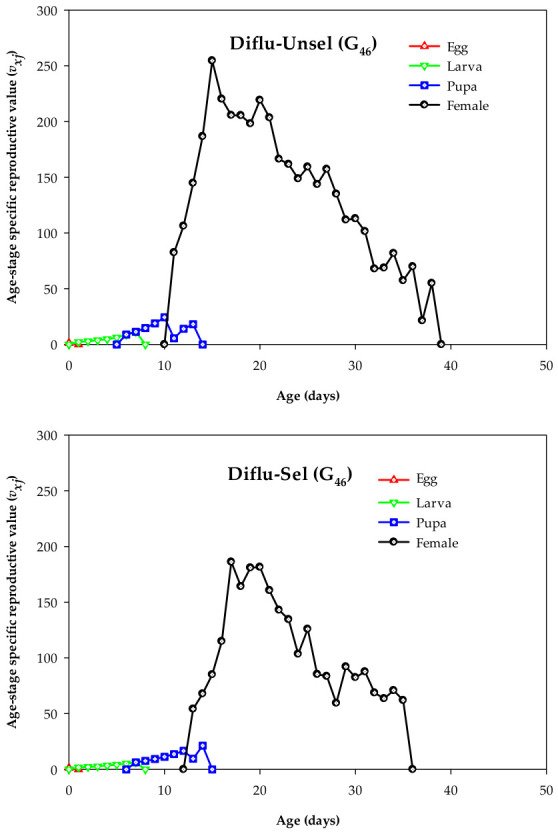
Age–stage reproductive values (*v_xj_*) in the diflubenzuron-selected (Diflu-Sel, G_46_) and diflubenzuron-unselected (Diflu-Unsel, G_46_) strains of *Musca domestica*.

The *v_xj_* value of *M. domestica*, which is used to predict population growth and reproduction rate, was significantly reduced in Diflu-Sel compared to Diflu-Unsel. Upon emergence of *M. domestica* females, the *v_xj_* values reached peaks at ages 15 (254.58 eggs/day) and 17 (186.15 eggs/day) in Diflu-Unsel and Diflu-Sel, respectively (Figure 4).

## 4. Discussion

Chemical insecticides remain the most effective tools for controlling medically and agriculturally important pests. Therefore, understanding the evolution, fitness costs, and mechanisms of resistance to novel chemical insecticides is vital for establishing rational guidelines for use and application [10,17,30,32]. Biorational insecticides, such as chitin synthesis inhibitors, are considered effective alternatives for managing crop pests and also have significant medical and veterinary relevance. Because of their relatively low mammalian toxicity, these biorational insecticides are generally regarded as safer for public health and the environment than conventional insecticides [2,63,64,65]. The efficacy of biorational insecticides, including diflubenzuron, has been well documented against *M. domestica* [2,12,13,64,65], *Stomoxys calcitrans* (L.) [65], *Cx. quinquefasciatus* [14,66], and *Aedes albopictus* Skuse [67].

Estimating realized heritability (*h*^2^) in accordance with quantitative genetic theory is one of the most reliable approaches for characterizing the evolutionary pattern of insecticide resistance in insect pests resulting from selection experiments [20,23]. In our study, the estimated *h*^2^ value for *M. domestica* after 42 generations of selection with diflubenzuron was 0.054, suggesting that resistance to diflubenzuron in *M. domestica* is influenced more by environmental factors than by additive genetic variation. Although resistance in the Diflu-Sel (G_46_) strain increased by ~320-fold in comparison to Diflu-Unsel (G_46_), the increase in resistance was only 7.5-fold when compared to Diflu-Unsel (G_5_) [10]. This indicates a slow rate of resistance development, consistent with the low estimated *h*^2^ value. Furthermore, the absence of a true laboratory-susceptible reference strain likely led to an underestimation of resistance levels and, consequently, the realized heritability.

Consistent with our findings, low *h*^2^ values were reported for resistance to fipronil (*h*^2^ = 0.05) [22], lambda-cyhalothrin (*h*^2^ = 0.06) [23], methoxyfenozide (*h*^2^ = 0.17) [25], and pyriproxyfen (*h*^2^ = 0.03) [24] in *M. domestica*. In contrast, substantially higher *h*^2^ values for resistance to spiromesifen (*h*^2^ = 0.59), clothianidin (*h*^2^ = 0.38), and spinosad (*h*^2^ = 0.68) resistance were observed in *M. domestica* [40,68,69], indicating a strong contribution of additive genetic variation and a potential for resistance evolution alleles under selection pressure. In the present study, the relatively low *h*^2^ value suggests a lower initial frequency of resistance alleles. Nevertheless, diflubenzuron should be incorporated into rotational insecticide programs to control *M. domestica*, delay resistance development, and preserve long-term efficacy.

In general, resistant strains often exhibit developmental and reproductive traits that are disadvantageous relative to susceptible strains [45]. In the present study, the Diflu-Sel strain exhibited a significantly longer larval duration than the Diflu-Unsel strain. Similarly, larval duration was significantly prolonged in fipronil-, cyproflanilide-, and fluxametamide-resistant strains of *M. domestica*, *Chilo suppressalis* (Walker), and *Spodoptera frugiperda* (J. E. Smith), respectively [33,70,71].

Feeding disruption, reduced appetite, altered metabolic processes, physiological developmental imbalance, and enhanced metabolic detoxification may contribute to the prolonged larval stage observed in diflubenzuron-exposed individuals [70,71,72]. However, extended larval development may indirectly enhance field-level management of *M. domestica* by increasing exposure to natural enemies, such as parasitoids and predators, and by forcing larvae to utilize suboptimal food resources to complete development, thereby reducing growth performance and reproductive output.

Additionally, the Diflu-Sel strain exhibited prolonged pupal and preadult durations, a shorter adult lifespan, reduced total longevity from egg to adult, fewer oviposition days, and lower fecundity than the Diflu-Unsel strain. These findings indicate that the fitness costs associated with diflubenzuron resistance are closely linked to these life-history traits, potentially facilitating resistance management when selection pressure is relaxed or temporarily removed.

In agreement with our results, adverse effects on comparable biological parameters have been reported in various insecticide-resistant strains, including *M. domestica* [33,37,39,40,73], *Ae. aegypti* [74], *D. koenigii* [45,46], *Oxycarenus hyalinipennis* (A. Costa) [75], *S. frugiperda* [71], *P. xylostella* [76], and *C. suppressalis* [70]. In contrast, egg duration, the adult preoviposition period (APOP), and the female ratio did not differ significantly between the Diflu-Sel and Diflu-Unsel strains of *M. domestica*.

Selection with diflubenzuron also significantly altered the reproductive and growth potential of *M. domestica*. Population traits (i.e., *r*, *λ*, *GRR*, and *R*_0_) serve as key indicators of the potential reproductive and growth rates of insect pests in a given environment. Alterations in these parameters can disrupt population growth and reproductive performance in resistant individuals [45]. Compared with the Diflu-Unsel strain, the *r*, *λ*, *R*_0_, and GRR values were markedly reduced, whereas the T and DT values of the Diflu-Sel strain were significantly prolonged. Moreover, the *m_x_*, *f_x_*, *s_xj_*, *l_x_m_x_*, *v_xj_*, and *e_xj_* values were consistently lower for the Diflu-Sel strain than for the Diflu-Unsel strain, indicating that the development of diflubenzuron resistance adversely affected survival and reproductive output in *M. domestica*. In agreement with our results, significant reductions in *r λ*, *R*_0_, GRR, *m_x_*, *f_x_*, *s_xj_*, *l_x_m_x_*, *v_xj_*, and *e_xj_* values have been reported in other insecticide-resistant pests, including *M. domestica* [33,34,38], *Sitobion miscanthi* (Takahashi) [77], *D. koenigii* [45,46], *C. suppressalis* [70], and *Ostrinia furnacalis* (Guenée) [31].

The combined reduction in *r*, *λ*, GRR, and *R*_0_, together with the prolongation of T and DT, is likely to result in a slower population growth rate of *M. domestica* following 42 generations of selection with diflubenzuron. In addition, the substantially reduced *R*_0_ of the Diflu-Sel strain resulted in a relative fitness value of 0.39. Similarly, reduced relative fitness values have been reported in *M. domestica* strains treated with fipronil (*Rf* = 0.13), lambda-cyhalothrin (*Rf* = 0.26), imidacloprid (*Rf* = 0.61), pyriproxyfen (*Rf* = 0.51), methoxyfenozide (*Rf* = 0.31), chlorantraniliprole (*Rf* = 0.34), clothianidin (*Rf* = 0.34), and alpha-cypermethrin (*Rf* = 0.50) [33,34,35,36,38,39,40,78].

In the present study, the development of diflubenzuron resistance is associated with significant disadvantages in the Diflu-Sel strain, likely reflecting trade-offs in resource and energy allocation. Such developmental and reproductive trade-offs may influence the evolutionary trajectory of resistance. Under continuous diflubenzuron selection pressure, the Diflu-Sel strain appears to require a substantial energetic investment to survive, thereby imposing fitness costs and leading to adverse developmental and reproductive consequences.

## 5. Conclusions

In conclusion, after 42 generations of selection, Diflu-Sel (G_46_) *M. domestica* developed 319.935-fold resistance to diflubenzuron compared with the Diflu-Unsel (G_46_) strain. Despite this high resistance level, Diflu-Sel (G_46_) *M. domestica* incurred substantial fitness and survival costs, as evidenced by less favorable life-history and population parameters relative to Diflu-Unsel (G_46_) *M. domestica*. These findings provide important insights for developing effective resistance management strategies targeting diflubenzuron resistance in *M. domestica*. Our results suggest that temporary withdrawal of diflubenzuron, or its rotational use with other effective new insecticides with different modes of action, may help restore susceptibility and prolong its field efficacy for the sustainable management of *M. domestica*. Additionally, integrated pest management approaches, including the judicious and limited use of chemical insecticides, biological control measures, and improved sanitation practices in and around livestock facilities and human dwellings, should be incorporated to achieve long-term and effective control of *M. domestica*.

## Figures and Tables

**Table 1:** Description of equations used for estimating population parameters of *Musca domestica*.

Parameter	Equation	Equation #	Description
Age-specific survival rate (*l_x_*)	$l_{x}=\sum_{j=1}^{m}s_{xj}$	(6)	Pooled probability that a newly laid egg will survive to age *x*. Because *lx* represents the pooled survival rates of all stages at age *x*, stage differentiation is not possible.
Age-specific fecundity (*m_x_*)	$m_{x}=\frac{\sum_{j=1}^{m}s_{xj}f_{xj}}{\sum_{j=1}^{m}s_{xj}}$	(7)	Average fecundity of all individuals at age *x*. *f_xj_* is the average fecundity of individuals at age *x* and stage *j*, and *m* is the number of stages.
Net reproductive rate (*R*_0_)	$R_{0}=\sum_{x=0}^{\infty }l_{x}m_{x}$	(8)	Total number of offspring that an individual can produce during its lifetime.
Intrinsic rate of increase (*r*)	$\sum_{x=0}^{\infty }{e^{-r(x+1)}l}_{x}m_{x}=1$	(9)	Population growth rate as time approaches infinity, with the population reaching a stable age–stage distribution, calculated by the Euler–Lotka equation [53,54] with age indexing set to zero [55].
Finite rate of increase (λ)	λ = *e^r^*	(10)	Population size will increase at a rate of λ per time unit.
Mean generation time (*T*)	$T=\frac{lnR_{0}}{r}$	(11)	Length of time that a population requires to increase its size in the stable age–stage distribution to *R*_0_-fold.
Life expectancy (*e_xj_*)	$e_{xj}=\sum_{\;i=x}^{\infty }\sum_{y=j}^{m}s_{iy}^{\prime }$	(12)	Expected duration of time that an individual of age *x* and stage *j* will survive after age *x*. $s_{iy}^{\prime }$ is the probability that an individual of age *x* and stage *j* will survive to age *i* and stage *y* [51].
Reproductive value (*v_xj_*)	$v_{xj}=\frac{e^{r(x+1)}}{s_{xj}}\sum_{i=x}^{\infty }e^{-r(i+1)}\sum_{y=j}^{k}s_{iy}^{\prime }f_{iy}$	(13)	*v_xj_* of an individual at age *x* and stage *j* to future offspring [56,57].
Relative fitness (*Rf*)	$Rf=\frac{R_{0}\;of\;Diflu-Sel\;(G_{46})}{R_{0}\;\;of\;Diflu-Unsel\;(G_{46})}$	(14)	Capability of an individual to survive and reproduce in comparison to other individuals of the same species [30].

**Table 2:** Resistance levels of *Musca domestica* selected with diflubenzuron under laboratory conditions.

Strain	LC_50_ (mg/L) ^a^	95% FLs ^b^	Fit of the Probit Line				RR (95% CL) ^c^
			Slope ± SE	*χ* ^2^	df	*p*	
Diflu-Unsel (G_46_)	0.021	0.017–0.026	2.83 ± 0.41	1.13	3	0.77	1
Diflu-Sel (G_46_)	6.824	5.193–9.613	1.95 ± 0.31	1.99	3	0.57	319.935 (222.690–459.646)

Diflu-Unsel = diflubenzuron-unselected strain; Diflu-Sel = diflubenzuron-selected strain. ^a^ Median lethal concentration expressed in milligrams per liter. ^b^ Fiducial limits. ^c^ Resistance ratio and confidence limits, which were calculated following Robertson et al. [59].

**Table 3:** Realized heritability (*h*^2^) of diflubenzuron resistance in the Diflu-Sel (G_46_) *Musca domestica*.

Insecticide	Initial LC_50_ (log) ^a^	Final LC_50_ (log) ^a^	G ^b^	*R* ^c^	*p* ^d^	*i* ^e^	Initial Slope	Final Slope	*σp* ^f^	*S* ^g^	*h* ^2 h^
Diflubenzuron	0.905 (−0.04) *	6.824 (0.83)	42	0.021	50	0.80	2.179	1.950	0.48	0.39	0.054

* Published by Hafez [10]. ^a^ Initial and final lethal concentration 50 values expressed in mg/L were determined for Diflu-Unsel (G_5_) and Diflu-Sel (G_46_), respectively. ^b^ Number of generations selected with diflubenzuron; G_1_–G_4_ generations were not selected with diflubenzuron. ^c^ Selection response. ^d^ The average survival during selection. ^e^ Selection intensity. ^f^ Phenotypic variance. ^g^ Selection differential. ^h^ Realized heritability of diflubenzuron resistance.

**Table 4:** Developmental durations and reproductive parameters of the Diflu-Unsel (G_46_) and Diflu-Sel (G_46_) strains of *M. domestica*.

Stage/Parameters	Diflu-Unsel (Mean ± SE)	Diflu-Sel (Mean ± SE)	95% CI Difference		*p*-Value
			Lower	Upper	
Larva (days)	5.16 ± 0.05 b	6.20 ± 0.04 a	0.91	1.17	<0.0001
Pupa (days)	5.55 ± 0.05 b	6.41 ± 0.05 a	0.72	1.00	<0.0001
Adult (days)	18.50 ± 0.81 a	10.48 ± 0.61 b	6.04	10.00	<0.0001
Preadult duration (days)	11.57 ± 0.06 b	13.59 ± 0.07 a	1.83	2.20	<0.0001
Total longevity from egg to adult (days)	30.07 ± 0.79 a	24.06 ± 0.59 b	4.08	7.93	<0.0001
Adult preoviposition period (APOP, days)	4.54 ± 0.19 a	4.35 ± 0.18 a	−0.33	0.70	0.47
Total preoviposition period (TPOP, days)	15.38 ± 0.18 b	17.77 ± 0.27 a	1.75	3.03	<0.0001
Oviposition period (days)	9.06 ± 0.55 a	5.92 ± 0.63 b	1.51	4.76	0.0003
Female ratio (%)	0.44 ± 0.05 a	0.38 ± 0.05 a	−0.08	0.20	0.37
Fecundity (eggs produced/female)	743.79 ± 69.43 a	265.19 ± 45.70 b	315.47	641.52	<0.0001

SE is the standard error, estimated via bootstrapping with 100,000 resamples. CI is the confidence interval. Diflu-Sel and Diflu-Unsel parameter differences were calculated via a paired bootstrap test at *p* < 0.05. Rows with different letters differ significantly from the Diflu-Unsel mean.

**Table 5:** Population parameters and relative fitness of the Diflu-Unsel (G_46_) and Diflu-Sel (G_46_) strains of *M. domestica*.

Parameters	Diflu-Unsel (Mean ± SE)	Diflu-Sel (Mean ± SE)	95% CI Difference		*p*-Value
			Lower	Upper	
Intrinsic rate of increase (*r*, day^−1^)	0.2535 ± 0.0090 a	0.1951 ± 0.0101 b	0.0322	0.0851	0.0001
Finite rate of increase (*λ*, day^−1^)	1.2885 ± 0.0115 a	1.2154 ± 0.0122 b	0.0404	0.1063	<0.0001
Generation time (T, days)	20.6689 ± 0.2312 b	22.0788 ± 0.3477 a	0.5831	2.2188	0.001
Doubling time (DT, days)	2.7348 ± 0.0989 b	3.5528 ± 0.1926 a	0.4110	1.2598	0.0017
Gross reproduction rate (GRR)	481.43 ± 77.46 a	270.76 ± 56.97 b	21.34	399.20	0.03
Net reproductive rate (*R*_0_, offspring per individual)	188.43 ± 31.55 a	74.25 ± 16.01 b	44.77	183.51	0.0014
Relative fitness (*Rf*)	1	0.39			

SE is the standard error, estimated via bootstrapping with 100,000 resamples. CI is the confidence interval. Diflu-Unsel and Diflu-Sel differences were calculated via a paired bootstrap test at *p* < 0.05. Rows with different letters differ significantly from the Diflu-Unsel strain.

## Data Availability

The raw data supporting the conclusions of this article will be made available by the authors on request.
